# A Personalized Ontology-Based Decision Support System for Complex Chronic Patients: Retrospective Observational Study

**DOI:** 10.2196/27990

**Published:** 2022-08-02

**Authors:** Esther Román-Villarán, Celia Alvarez-Romero, Alicia Martínez-García, German Antonio Escobar-Rodríguez, María José García-Lozano, Bosco Barón-Franco, Lourdes Moreno-Gaviño, Jesús Moreno-Conde, José Antonio Rivas-González, Carlos Luis Parra-Calderón

**Affiliations:** 1 Computational Health Informatics Group, Institute of Biomedicine of Seville Virgen del Rocío University Hospital, Consejo Superior de Investigaciones Científicas University of Seville Seville Spain; 2 Primary Care Camas Clinical Management Unit Seville Spain; 3 Internal Medicine Department Virgen del Rocío University Hospital Seville Spain

**Keywords:** adherence, ontology, clinical decision support system, CDSS, complex chronic patients, functional validation, multimorbidity, polypharmacy, atrial fibrillation, anticoagulants

## Abstract

**Background:**

Due to an increase in life expectancy, the prevalence of chronic diseases is also on the rise. Clinical practice guidelines (CPGs) provide recommendations for suitable interventions regarding different chronic diseases, but a deficiency in the implementation of these CPGs has been identified. The PITeS-TiiSS (Telemedicine and eHealth Innovation Platform: Information Communications Technology for Research and Information Challenges in Health Services) tool, a personalized ontology-based clinical decision support system (CDSS), aims to reduce variability, prevent errors, and consider interactions between different CPG recommendations, among other benefits.

**Objective:**

The aim of this study is to design, develop, and validate an ontology-based CDSS that provides personalized recommendations related to drug prescription. The target population is older adult patients with chronic diseases and polypharmacy, and the goal is to reduce complications related to these types of conditions while offering integrated care.

**Methods:**

A study scenario about atrial fibrillation and treatment with anticoagulants was selected to validate the tool. After this, a series of knowledge sources were identified, including CPGs, PROFUND index, LESS/CHRON criteria, and STOPP/START criteria, to extract the information. Modeling was carried out using an ontology, and mapping was done with Health Level 7 Fast Healthcare Interoperability Resources (HL7 FHIR) and Systematized Nomenclature of Medicine Clinical Terms (SNOMED CT; International Health Terminology Standards Development Organisation). Once the CDSS was developed, validation was carried out by using a retrospective case study.

**Results:**

This project was funded in January 2015 and approved by the Virgen del Rocio University Hospital ethics committee on November 24, 2015. Two different tasks were carried out to test the functioning of the tool. First, retrospective data from a real patient who met the inclusion criteria were used. Second, the analysis of an adoption model was performed through the study of the requirements and characteristics that a CDSS must meet in order to be well accepted and used by health professionals. The results are favorable and allow the proposed research to continue to the next phase.

**Conclusions:**

An ontology-based CDSS was successfully designed, developed, and validated. However, in future work, validation in a real environment should be performed to ensure the tool is usable and reliable.

## Introduction

### Chronic Diseases and Multimorbidity

Due to an increase in life expectancy, the prevalence of chronic diseases is also rising [1]. According to the World Health Organization (WHO), chronic diseases are health problems requiring treatment over years or decades [2]. The diagnosis of 2 or more chronic conditions in a patient at the same time is called multimorbidity [3]. Patients with multimorbidity, called complex chronic patients (CCPs), are characterized by being fragile, with polypharmacy, of older age, in emergency departments frequently, and having a higher rate of hospital readmissions [4]. CCPs also constitute a challenge for the health system, particularly for health professionals, as there are few specific guidelines for providing integrated health care [5].

### Polypharmacy

The increase in the number of CCPs is linked to a higher incidence of polypharmacy [6], defined as a patient taking 5 or more drugs [7]. The drugs offer clinical benefits and risks, but the complexity of CCPs makes this group more susceptible to errors [8].

Polypharmacy is associated with increased adverse reactions, reduced adherence to treatment, and increased demand for health care resources [9]. Currently, clinical practice guidelines (CPGs) recommend drugs for disease management based on clinical trials for specific diseases. However, CPGs do not consider the complications that can arise in a CCP due to interactions between different drugs for different diseases [10].

All this highlights the need for personalization of care and prescriptions due to each patient's specific situation [11].

### Clinical Guidelines

CPGs are defined as “systematically developed statements to assist practitioner and patient decisions about appropriate health care for specific clinical circumstances” [12]. Some CPGs provide recommendations for good interventions regarding different chronic diseases. However, there is lack of implementation of these CPGs in clinical practice due to different factors, such as subjective interpretation by health care professionals, the lack of time for health care professionals to read these CPGs [13], and clinical inertia [14], among others. In addition, a study carried out by the National Institute of Health and Care Excellence (NICE) compared 12 CPGs and detected numerous dangerous interactions between the different recommendations given by each CPG [15].

Today, health care systems face the challenge of providing a new care model integrating all the CPGs [10] that do the following: take into account the individual situation of each patient; identify possible interactions between drugs prescribed to the same patient to avoid complications; consider the patients' life expectancy as, in some situations, prescribing the drug will not provide significant benefit to the patient; and if life expectancy is low, recommend beneficial and effective but less aggressive treatments.

### Clinical Decision Support Systems

Clinical decision support systems (CDSSs) are handy tools in the health framework and have 3 requirements [16]: (1) computable biomedical knowledge, (2) information about the specific situation of each patient, and (3) reasoning mechanisms to be able to provide personalized recommendations to each patient.

However, even with years of research in this field, health care professionals' acceptance of CDSSs is not satisfactory. This is one of the reasons why 95% of CDSSs are discarded [17]. Different studies have been conducted to determine what needs to be fulfilled during the development of the CDSS to be well accepted in the health care environment and especially by health care professionals. Shortlife et al [18] reported that biomedical informaticians had identified the following characteristics necessary to achieve good acceptance of a CDSS: users, in this case, health care professionals, must understand the basis and reasoning behind the CDSS recommendations; the CDSS developed in the health care environment should be intuitive and easy to use; the CDSS should support the health care professional by providing advice but always respecting their experience and knowledge; and the recommendations provided by the CDSS should be evidence-based and offer resources (scientific papers, CPGs, studies, etc) to review the validity of those recommendations.

Trinkley, Blakeslee, et al [19] explored the beneficial features of a CDSS for primary care from the health professional's perspective. They concluded that the design of the CDSS should be user-centered and take into account the user's needs in terms of content, presentation, and functionality and that the CDSS should be personalized to the health professional's needs, providing relevant information and optimizing the workflows.

Trinkley, Kahn, et al [17] highlighted the importance of reducing alerts to a minimum to minimize health professional fatigue, including all health care team members in the workflow, and encouraging health professionals to follow the recommendations. The latter point focuses on time savings during health care, such as ordering a test directly from the CDSS or updating the medical record with the data entered in the CDSS automatically.

### Ontology-Based Systems

Studies such as that by Van de Velde et al [20] demonstrate that integrating the knowledge contained in CPGs and other knowledge sources into a CDSS improves clinical practice. To carry out this integration, it is necessary to make an intermediate step that models this information's content [21]. One way to perform this intermediate step and, in this way, to infer the specific clinical knowledge, is by designing an ontology.

An ontology is an excellent way to organize existing knowledge in CPGs [22]. These kinds of resources define a common vocabulary that allows researchers to share information about the same field [23]. Borst [24] defines ontology as “an explicit and formal specification of a shared conceptualization.” According to Lekhchine [25], each element of the definition of ontology provided by Borst should be understood as follows: explicit specification—each ontology concept and associated features are defined in a declarative form; formal—this allows ontologies to be interpretable by a machine; shared—here, the knowledge that is shared in the ontology is consensual; and conceptualization—this involves linking to the abstraction of a phenomenon by identifying concepts related to that phenomenon.

Konaté et al [22] identified the most critical needs for the decision to develop an ontology to be the following: a shared understanding between different software developers; reuse of knowledge in a particular domain; helping the different actors in a domain to understand each other better and increase their knowledge; distinguishing 2 types of knowledge, operational knowledge and domain-focused knowledge; and analyzing existing knowledge about a domain.

With consideration to all the characteristics analyzed in this section and to provide personalized and integrated care to CCPs, the PITeS-TIiSS (Telemedicine and eHealth Innovation Platform: Information Communications Technology for Research and Information Challenges in Health Services) tool has been designed, developed, and validated.

The main objective of the PITeS-TIiSS project is to improve evidence-based decision-making capacity and reduce variability in clinical practice in the domain of integrated care of CCPs through the use of advanced semantic interoperability and clinical decision support methods and tools.

## Methods

### Ethics Approval

This study obtained authorization from Virgen del Rocío University Hospital (VRUH) review board chaired by Victor Margalet on November 24, 2015 (number PI15/01213).

### Selecting the Study Scenario

For the development and clinical validation of the PITeS-TIiSS tool, a specific study scenario was defined.

To design this study scenario, the prevalence of chronic diseases was taken into account in the health area where this clinical validation was carried out. This ensured the recruitment of patients for the study and the high availability of information recorded in the electronic health record (EHR).

The ultimately chosen scenario focused on treatment with anticoagulant drugs in patients with atrial fibrillation. This pathology has a high prevalence, and an early treatment protocol needs to be established due to the complications it can cause [26].

The study was conducted in the Andalusian Health Service in Andalusia, a Spanish region with more than 8 million inhabitants. Specifically, the study area was located at the VRUH in Seville and the Primary Care Center in Camas (Seville).

### Study, Analysis, and Selection of Knowledge Bases

#### Selection Criteria

Once the study setting was defined, the clinical researchers analyzed the evidence-based knowledge bases most commonly used in clinical practice concerning atrial fibrillation, care of CCPs, and oral anticoagulant therapy. More than 20 clinical documents with relevant information were identified. Several criteria were also considered in the selection of clinical records to be used for knowledge extraction: the frequency of application in clinical practice of the information included in the document; the level of updating of the clinical data; the level of consensus of the clinical data; and the clinical documents included in the clinical action protocols of the VRUH and its affiliated centers, where the PITeS-TIiSS tool was to be validated.

After the analysis of the identified clinical documents, it was decided to select the following sources of knowledge.

#### STOPP/START Criteria

These criteria were first published in Ireland in 2008 and were updated in 2014 [27]. This knowledge base focuses on describing the most common errors in drug prescription. One of their most important features is that these criteria can be integrated into computer systems [28]. This knowledge base is used at the European level to analyze drugs prescribed to older adult patients. These criteria aim to control the polypharmacy to which this type of patient is usually subjected in order to optimize the drugs prescribed and prevent side effects that cause major complications. This document makes it possible to study each patient's individual and specific situations and obtain personalized recommendations for these situations. Moreover, it provides 2 types of recommendations: (1) prescription of drugs necessary for each patient according to the patient’s condition; and (2) deprescription of drugs whose effect, due to the particular situation of each patient, may be more harmful than beneficial. These criteria have been demonstrated to detect potentially dangerous and inappropriate prescriptions and improve the quality of prescriptions.

#### LESS-CHRON Criteria

These criteria guide the optimization of drug prescription in patients with chronic diseases and have been developed using the Delphi method. This document analyzes different pharmacological characteristics, including the indications for which the drug is prescribed, conditions that recommend deprescribing the drug, health variables to monitor the behavior of the drug, and the time that should elapse between different follow-ups. Based on these characteristics, a list of 27 criteria has been compiled [29].

#### PROFUND Index

The PROFUND Index is a scale designed by Spanish physicians and is widely used in Spain. It is a prognostic index for CCPs. It includes different types of variables: demographic, clinical, laboratory, functional, socio-familial, and care [30].

#### Clinical Practice Guidelines

CPGs are systematically developed guidelines to assist health professionals in developing care to provide personalized care for individual patients in specific clinical circumstances [13]. Clinical researchers recommended using a CPG on the diagnosis and treatment of atrial fibrillation [31].

### Knowledge Extraction and Decision Rule Design

After selecting the knowledge bases, 2 researchers (ERV and CAV) specializing in medical informatics extracted the relevant information for the defined study scenario. Due to the length of some of the documents, information was searched using keywords related to anticoagulant and antiplatelet drugs, such as “oral anticoagulants,” “anticoagulation,” “anticoagulants,” “vitamin K antagonists,” “antiaggregants,” “antiaggregation,” “acetylsalicylic acid,” and “clopidogrel.”

The information extracted from the selected documents was collected in a tabulated record in the form of rules. The clinical researchers (BBF, MJGL, and LMG) validated these before continuing with the procedure.

The extracted rules have a specific structure. Clinical concepts are the variables that are associated with the values (numeric, Boolean, and others), for example, age, atrial fibrillation, antiaggregants, etc. Premises are the values and features that are associated with clinical concepts, for example, greater than or less than, true or false, and number.

Clinical statements include the text that makes the recommendation to be shown to the health professional, for example “Deprescribe in any case.”

Likewise, 2 types of rules have been defined: mini-rules are rules that relate to a single clinical concept and a single premise associated with a clinical statement, for example “IF Treatment with Ticlopidine THEN Deprescribe in any case”; super-rules are those that relate to several clinical concepts and their premises to a clinical statement. for example “IF Atrial Fibrillation AND (Mitral Stenosis OR Mechanical Heart Valve) THEN Do Not Prescribe New Oral Anticoagulants (Apixaban, Dabigatran, Edoxaban, and Rivaroxaban).”

A total of 59 clinical recommendations related to the prescription and deprescription of drugs were extracted in the clinical setting of patients with atrial fibrillation. The clinical recommendations were displayed in a personalized way in the CDSS developed based on the information collected for each patient.

### Information Modeling and Mapping

With all the necessary information collected in the tabulated document, the ontology was defined. To implement the clinical concepts, the premises, clinical statements, and the relationships between them, Protégé software was used.

**Figure 1:**
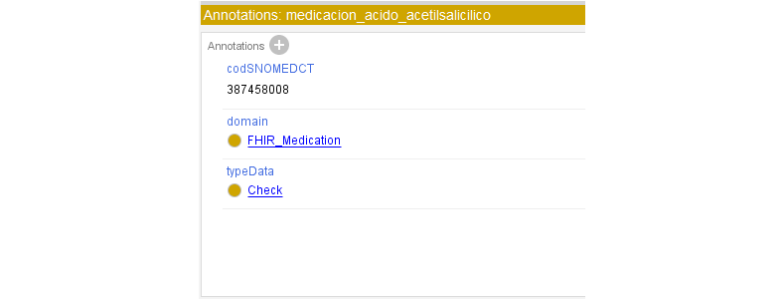
Systematized Nomenclature of Medicine Clinical Terms (SNOMED CT) and Health Level 7 Fast Healthcare Interoperability Resources (HL7 FHIR) mappings.

Initially, the clinical concepts were added and mapped through annotations with the Health Level 7 Fast Healthcare Interoperability Resources (HL7 FHIR) standard [32] to facilitate syntactic interoperability and with Systematized Nomenclature of Medicine Clinical Terms (SNOMED CT) [33] to facilitate semantic interoperability (Figure 1).

Specifically, Figure 1 is about the drug acetylsalicylic acid. Overall, 100% of the ontology concepts were mapped with FHIR, and 52% of the concepts were mapped with SNOMED CT. It was not possible to map all the concepts with SNOMED CT, as some of these concepts do not exist within that terminology.

Once the clinical concepts were introduced, the premises and clinical statements were implemented. The ontology's internal logic was established through the use of object properties, data properties, and the relationships between the concepts.

**Figure 2:**
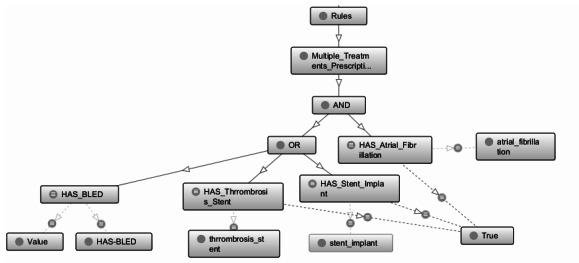
Super-rule example. HAS: Hypertension, Abnormal Renal/Liver Function, Stroke; HAS-BLED: Hypertension, Abnormal Renal/Liver Function, Stroke, Bleeding History or Predisposition, Labile INR, Elderly, Drugs/Alcohol Concomitantly.

The establishment of the relationships between the clinical concepts and the premises to design the mini-rules was carried out through a propositional logic system (true or false, less than or greater than, etc). For the super-rules' design (Figure 2), the logical relationships between the different clinical concepts and premises (and/or) was also established.

Figure 2 shows an example of the super-rule related to the National Institute of Health Stroke Scale (NIHSS). In this case, for the rule to be fulfilled, the patient must necessarily have multiple prescribed treatments and atrial fibrillation and additionally have 1 or more of the following characteristics: a certain value on the HAS-BLED (Hypertension, Abnormal Renal/Liver Function, Stroke, Bleeding History or Predisposition, Labile INR, Elderly, Drugs/Alcohol Concomitantly) score on bleeding risk, an implanted stent, or a thrombosis in a stent.

### Design and Development of the CDSS

For the design and development of the CDSS, including the user interface, the ITCBio (Infrastructure for Translational and Clinical Research based on Standardization, Integration, Advanced Analytics) infrastructure [34] was used. This is a computer tool based on free software that has been developed to provide support for research in Andalusia (Spain).

**Figure 3:**
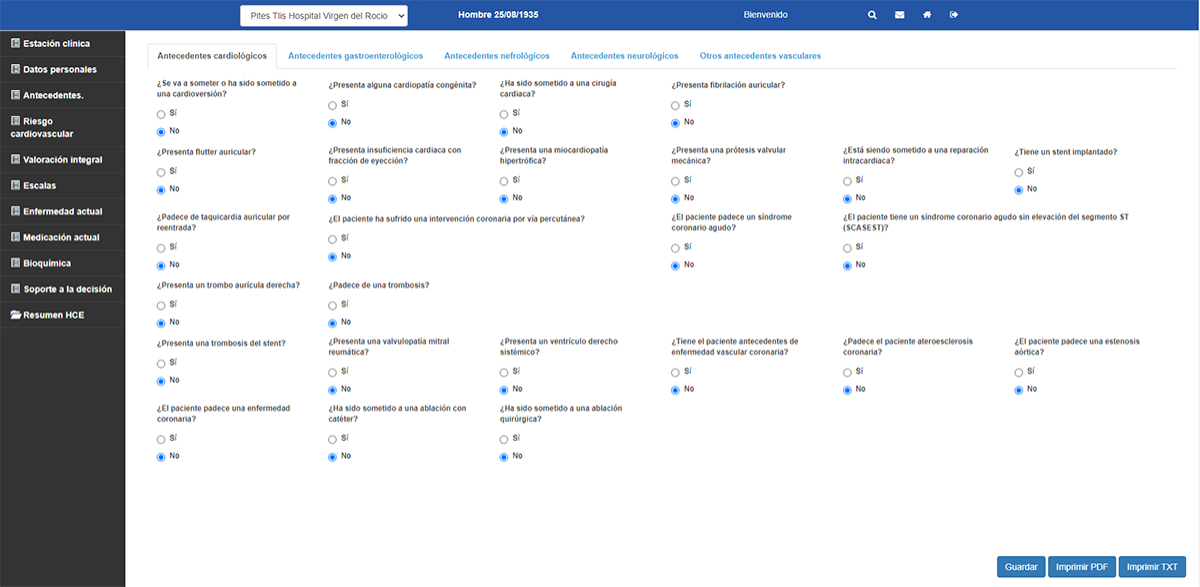
Tool interface.

First, the technical developer technicians (JMC, JARG, and GAER), together with the researchers specializing in medical informatics (ERV and CAR), completed the user interface design. Based on the tabulated document, the questions that are shown to the health professionals to collect information on each patient were formulated. In order to make the tool usable and speed up the collection of information, the questions are distributed in different sections related to the patient's anamnesis, exploration, complementary tests, and treatment (Figure 3). Based on each premise, a question is formulated and presented to the health professionals through the tool's interface. Thus, depending on the answers selected by the health professionals, the premises may or may not be fulfilled, and the recommendations whose premises are fulfilled are then executed.

Second, for the integration of the ontology containing the ITCBio infrastructure rules, the free software integration engine Mirth Connect was used, whose function is focused on the integration of tools in the health care field. One of this integration engine's most important functions is that it natively provides the following HL7 messaging standards. This integration engine allows interoperating with the clinical information of the recruited patients present in the EHR through the development of specific channels.

Once the health professional enters the information into the CDSS, ontology queries are made, rules are executed, and recommendations related to the pharmacological prescription are generated all through the Mirth Connect integration engine.

**Figure 4:**
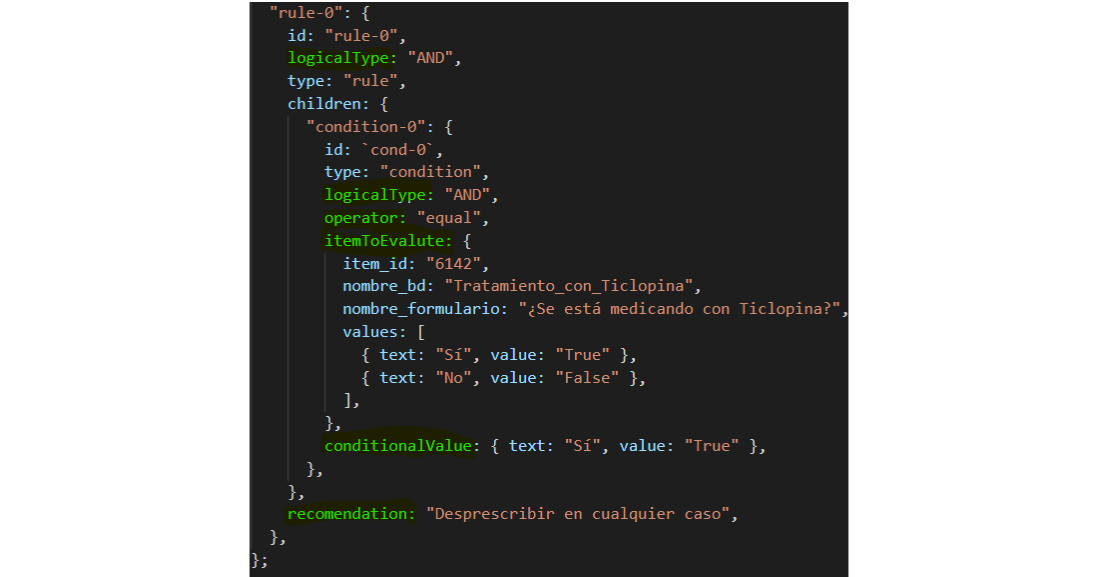
Mini-rule.

Figure 4 shows an example of the information used by the Mirth Connect integration engine after querying the ontology. In this case, it is a mini-rule in which the clinical concept corresponds to the attribute “name_bd” and is “Ticlopidine treatment.” The Premise corresponds to the attribute “values” and, in this case, has the value “TRUE.” The Clinical Statement corresponds to the attribute “recommendation,” and its value in this example is “Deprescribe in any case.”

**Figure 5:**
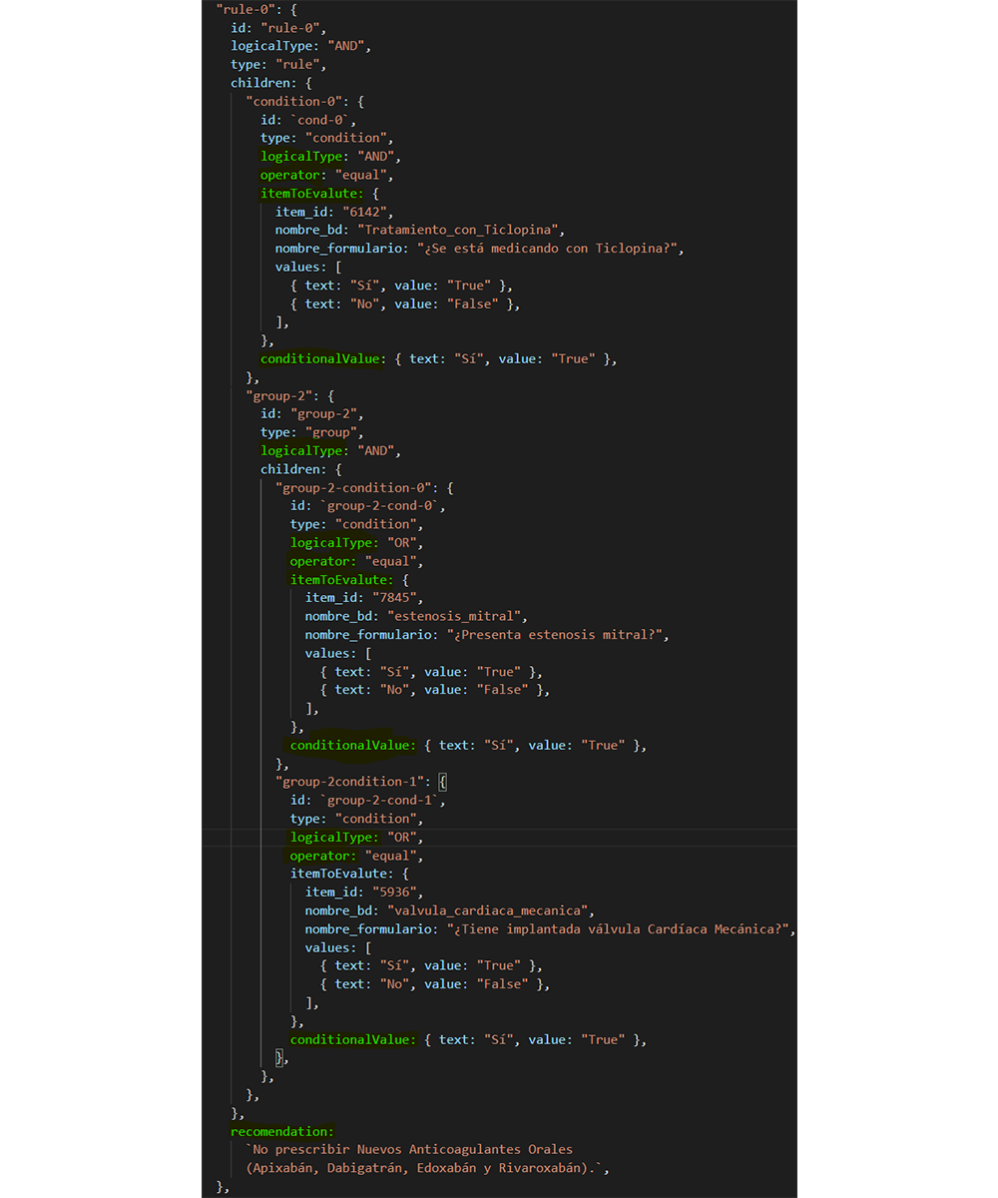
Super-rule.

Figure 5 shows an example of the information used by Mirth Connect concerning a super-rule. In this case, 3 clinical concepts can be observed. One of them must have the value “TRUE,” and the other 2 are nested, and only 1 of them must have the value “TRUE” for the recommendation to be presented.

In this way, 3 types of information are shown to health professionals: (1) recommendations on prescribing, (2) recommendations on deprescription, and (3) recommendations that have not been implemented due to a lack of information.

To check that the rules have been executed correctly, each recommendation contains a drop-down list showing the assumptions that have been taken into account to execute the rule. Additionally, health professionals are shown information on the clinical concepts and premises that have been fulfilled to execute the rule and display the recommendation, the CPG from which the recommendation has been extracted, the page and section of the CPG where the recommendation appears, and the level of evidence of the recommendation.

Moreover, for those rules that are not executed due to a lack of information, the CDSS simulates the result that would be generated if that value had been completed, fulfilling the premise and not fulfilling it. In this way, the tool analyzes whether the result would be affected by fulfilling these 2 hypotheses, and the rule would be executed.

### Clinical Validation

For patient recruitment and subsequent clinical validation, a number of inclusion criteria were defined: 65 years old or older, polypharmacy (5 or more drugs daily [7]), multimorbidity (with at least 2 chronic diseases or comorbidities in the multimorbidity classification [4]), with atrial fibrillation, and undergoing oral anticoagulants treatment. Meanwhile, the exclusion criterion was as follows: patients at the end of life with a short-term prognosis of less than six 6 months.

## Results

Once the PITeS-TIiSS tool was designed and developed, 2 different tasks were carried out to test the functioning of the tool. First, retrospective data from a real patient who met the inclusion criteria were used. Second, the analysis of an adoption model was performed through the study of the requirements and characteristics that a CDSS must meet in order to be well accepted and used by health professionals.

### Functional Validation

For the functional validation, a patient was identified from the Camas Health Center in Seville, Spain, who met the inclusion criteria.

After this, the process of extracting knowledge from the EHR and generating personalized recommendations was carried out. This process of extracting clinical information consisted of 3 phases: (1) In the first stage, patient's identification data were included in the ITCBio platform; once the patient was included, the ITCBio platform extracted the information on hospitalizations, prescriptions, and other patient data useful for the study. (2) In the second stage, the tool had not been tested in a real environment, and retrospective data were used for functional validation; these data were extracted from the EHR of the patient chosen by a clinical researcher (ERV) and entered into the PITeS-TIiSS tool; this was obtained from the date on which the patient was diagnosed with atrial fibrillation and prescribed medication. (3) In the third phase, based on the data entered into the PITeS-TIiSS tool, the rules whose assumptions were met were executed, and personalized recommendations on the prescription of medicines were generated.

As seen in Textbox 1, patient 0 meets the first, third, and fourth inclusion criteria, as he is over 65 years old, has multimorbidity, and has been diagnosed with atrial fibrillation.

He also met the inclusion criteria related to polypharmacy and prescription anticoagulants (Textbox 2).

Once this patient's information was entered into the PITeS-TIiSS tool, including the results of the Pfeiffer Questionnaire, PROFUND Index, and CHA_2_DS_2_-VASc (congestive heart failure, hypertension, age ≥75 years, diabetes, stroke, vascular disease, age 65 to 74 years, and sex category), 9 personalized recommendations were displayed (Textbox 3).

For each recommendation shown for this clinical case (Textbox 3), the following assumptions have been met: recommendation 1, the patient has atrial fibrillation and is prescribed a vitamin k antagonist; recommendation 2, the patient has atrial fibrillation and is prescribed antiaggregants and oral anticoagulants; recommendation 3, the patient has atrial fibrillation; recommendation 4, the patient has atrial fibrillation and arterial hypertension and is prescribed oral anticoagulants; recommendation 5, the patient has atrial fibrillation and is prescribed oral anticoagulants; recommendation 6, the patient has atrial fibrillation and is prescribed oral anticoagulants; recommendation 7, the patient has atrial fibrillation and is prescribed oral anticoagulants; recommendation 8, the patient is prescribed a vitamin k antagonist; recommendation 9: the patient has atrial fibrillation and is prescribed antiaggregants and oral anticoagulants.

**Patient 0.**

**Patient 0**

Gender: Male

Age: 70 years

Pathologies:

- Dyslipidemia
- Obesity
- Arterial hypertension
- Diabetes Mellitus 2
- Atrial fibrillation
- Asthma
- Chronic obstructive pulmonary disease
- Mild nonproliferative retinopathy
- Prostatic adenocarcinoma.

**Prescribed drugs.**

**Prescribed drugs**

- Treatment for type 2 diabetes mellitus: rapid and mixed insulin and metformin
- Treatment for arterial hypertension: irbesartan, hydrochlorothiazide and amlodipine
- Analgesic treatment with paracetamol
- Treatment for prostate cancer with decapeptyl, tamsulosin hydrochloride, and bicalutamide
- Antiaggregant treatment with acetylsalicylic acid
- Anticoagulant treatment with aldocumar
- Lipid-lowering treatment with atorvastatin
- Omeprazole stomach protector

**Personalized recommendations for patient 0.**

**Personalized recommendations displayed by the PITeS-TIiSS tool**

1. “It is recommended to keep the therapeutic range time (TRT) as high as possible.”
2. “It is recommended to avoid the combination of antiaggregants and anticoagulants if there is no other indication for platelet inhibition.”
3. “Antiplatelet therapy in monotherapy is not recommended for the prevention of stroke in patients with atrial fibrillation.”
4. “Blood pressure should be monitored closely in this patient.”
5. “Moderate alcohol consumption is recommended, as there is a risk of bleeding.”
6. “It is recommended that renal function be evaluated once a year.”
7. “It is recommended to thoroughly review the prescription, eliminating drugs that may cause bleeding and shortening prescription times.”
8. “It is recommended to reduce the dose of vitamin K antagonists if the patient is taking amiodarone.”
9. “It is recommended to deprescribe ASA [acetylsalicylic acid] as it is of no benefit.”

Following the retrospective validation of the tool, a health professional from a primary care center performed a thorough analysis of the results.

First, the health professional reviewed the EHR of the selected patient and checked that the data entered in the PITeS-TIiSS tool were correct.

Subsequently, the recommendations displayed for this patient were reviewed. As indicated in the section on the development methodology of the tool, the assumptions that execute each rule and show each recommendation can be checked by reviewing a drop-down that shows them.

After reviewing the assumptions, the health professional reviewed the displayed recommendations and the additional information in the CPGs that support each recommendation.

The health professional concluded that she would comply with all the recommendations provided.

### Adoption Model

Taking into account the analysis of the needs that a CDSS must meet to be well accepted that was carried out in the Introduction section, an adoption model was designed, consisting of an analysis of the 8 requirements that the PITeS-TIiSS tool must meet and how it can do so:

1. Health professionals need to understand the basis and reasons why the CDSS makes the relevant recommendations: the tool provides below each recommendation the premises fulfilled for that recommendation to be displayed. Thus, the health professional can quickly recognize which items are related to each recommendation and why it is displayed.

2. CDSS should be intuitive and easy to use (usability). For the design and development of the PITeS-TIiSS tool, the ISO 13407:1999 standard has been followed. Human-centered design processes are required for interactive systems, as this complies with the 4 following principles of user-centered design contemplated in this standard: (1) active involvement of users as specified in point 5; (2) appropriate assignment of roles to the PITeS-TIiSS tool and the user—different roles have been established to access other tool modules, such as the developer role and health professional role; (3) iterative design solutions—during the tool's design and development stage, meetings were held between the development team and the health professionals to achieve a tool adapted to their needs, and the rules were also revalidated by presenting different clinical cases in these meetings; (4) multidisciplinary design—the PITeS-TIiSS tool has been designed and developed by technical developers and clinical informatics researchers (JMC, JARG, GAER, ERV, and CAR) who have provides their clinical and informatics vision.

3. The CDSS should support the health professional and offer advice while respecting his or her experience and knowledge: the PITeS-TIiSS tool's recommendations are not presented as mandatory. In the interface where the recommendations are displayed, the health professional can indicate which rules will be followed and which rules are considered not to be followed. In turn, the health professional can justify why he or she has decided not to follow a specific recommendation. The latter allows the CPGs to be compared with actual clinical practice and to include changes in the CPGs that can improve clinical practice.

4. Recommendations should be evidence-based and provide resources to review the validity of these recommendations: in this case, the PITeS-TIiS tool offers the option of displaying in a PDF format the reasons why the recommendation has been shown. It shows the clinical concepts, the premises that have been met, the CPG from which this information has been extracted, the text in which the recommendation is found, the page and section where the recommendation is located, and the level of evidence of the recommendation.

5. The CDSS should be user-centered, taking into account the user's needs. Numerous meetings were held with the team of hospital and primary care health professionals throughout the design and development process. In these meetings, in addition to validating the content of the rules and recommendations, the health professionals' needs in terms of the development of the tool's interface were taken into account.

6. Fatigue-inducing alerts should be minimized. The PITeS-TIiSS tool does not display alerts or pop-up windows. Once the workflow has been completed and all the necessary patient information has been entered, the tool displays the personalized and justified recommendations.

7. All team members and workflow should be included in the tool's use. In this case, the PITeS-TIiSS tool focuses on health professionals—mainly internists, primary care physicians, and nurses.

8. Health professionals should be encouraged to use the tool. This feature is mainly about facilitating health professionals' work. It focuses on the need for a CDSS to connect to the EHR and primarily share information. The PITeS-TIiSS tool does not have a connection to the EHR. However, in the new Smart-PITeS project grant (#PI18/00700; Learning Health System for the Integrated Care and Adherence Management of Complex Chronic Patients), which is a continuation of the PITeS-TIiSS project, it is intended to connect with the EHR.

## Discussion

### Principal Findings

A personalized ontology-based CDSS called PITeS-TIiSS tool was designed and developed. A first functional validation was carried out to test and evaluate the proper functioning of this tool. A basic model for adopting the technology was designed by analyzing the needs of a CDSS to encourage its successful adoption in the health care field. These needs were extracted from different scientific publications that deal with this subject in-depth and have been added as references in this paper in the Introduction section.

The PITeS-TIiSS project is the third phase of a network of collaborative research projects whose overall objective is to provide a secure, open, and interoperable digital ecosystem to facilitate the design, development, validation, and implementation of telehealth service innovations. Specifically, this third phase aims to incorporate advanced semantic interoperability and clinical decision support tools into clinical practice to provide integrated and personalized care to CCPs.

Concerning functional validation, the results have been favorable and allow the research proposed for the fourth phase of this network of research collaboratives projects, called Smart-PITeS, to continue.

Moreover, a new front is emerging concerning different CPGs. This is because there may be inconsistencies between each CPG's recommendations, and, thanks to the information provided by health professionals on the reasons why they choose not to follow a specific recommendation, changes in the CPGs based on health care can be considered.

### Limitations

Although the research results are favorable, we intend to address several limitations during the completion of the Smart-PITeS project. Regarding the knowledge bases, those used to design the decision rules are those most commonly used in the VRUH health care area. It may happen that, in any other health care area, other CPGs are used. However, the established methodology can be applied to the design of new decision rules.

Due to many existing drugs and pathologies, to facilitate development and clinical validation, it was decided to study a specific scenario. In this case, it was CCPs with atrial fibrillation and prescribed oral anticoagulants. However, it is necessary to broaden the study of pathologies and drugs to provide real integrated care. In addition, the validation was carried out with a single clinical case, which does not conclusively ensure that the tool works correctly. Moreover, the validation of the PITeS-TIiSS tool was carried out with retrospective data. However, this potential limitation to prospective validation is expected to be addressed by the Smart-PITeS project currently underway and continuing this research approach. Finally, a lack of integration with the EHR has been identified, which means that one of the needs for the successful adoption of the health care field tool is not being met. One of the objectives of the Smart-PITeS project is to remedy this limitation. 

### Comparison With Prior Work

CDSS are useful tools for clinical practice. This is why there is a wide variety of proposals for such tools.

In Velickovski et al's [35] work in the field of CDSS for CCPs, they propose a CDSS focused on diagnosis and prevention in patients with chronic obstructive pulmonary disease. However, in this research, they did not use ontology as information modeling. Furthermore, they focused on a single pathology. Böttiger et al [36] also did not use ontology for the development of their CDSS. Instead, they focused on preventing and avoiding drug-drug interactions and adverse effects. Zhang et al [37] did use ontology as the main element of development. Their proposal focused on the diagnosis, assessment, and treatment of patients with type 2 diabetes mellitus. The difference between this proposal and the PITeS-TIiSS tool is that the first focuses on a single chronic disease, while the proposed tool aims to address numerous chronic pathologies. Beyond this, PITeS-TIiSS reports the assumptions that have been met for the personalized recommendations to be displayed. Konaté et al [22] proposed the development of a decision support system using an ontology. However, the field of development was not health care. The CDSSs proposed by Chen et al [38] and Madhusanka et al [39] were also based on an ontology. However, they again focused on a single pathology, such as type 2 diabetes mellitus 2. Bouaud et al [40] also proposed a CDSS and relied on an ontology for development. In this case, the researchers focused only on primary breast cancer patients. Finally, Miñarro-Giménez et al [41] developed an ontology-based CDSS focused on decision support for health professionals by providing pharmacological data to both professionals and patients. This tool is specifically focused on patients with genetic disorders to prevent adverse reactions or drug interactions in these patients. They concluded that this tool could be useful for personalized medicine.

### Conclusions

An ontology-based CDSS was successfully designed, developed, and validated. However, it has only been validated with retrospective information from 1 case study, so a more robust validation would be necessary to ensure that the tool is usable and reliable.

The next phase, the Smart-PITeS project, aims to overcome the limitations found in this project and also to ensure that the tool performs automatic learning and predictive models in relation to care and adherence to treatment of CCPs.

## Abbreviations

CCP: complex chronic patient
CDSS: clinical decision support system
CHA2DS2-VASc: congestive heart failure, hypertension, age ≥75 years, diabetes, stroke, vascular disease, age 65 to 74 years, and sex category
CPG: clinical practice guideline
EHR: electronic health record
ERDF: European Regional Development Fund
HAS-BLED: Hypertension, Abnormal Renal/Liver Function, Stroke, Bleeding History or Predisposition, Labile INR, Elderly, Drugs/Alcohol Concomitantly
HL7 FHIR: Health Level 7 Fast Healthcare Interoperability Resources
ITCBio: Infrastructure for Translational and Clinical Research Based on Standardization, Integration, Advanced Analytics
ITEMAS: Innovation in Medical Technologies and Health
NICE: National Institute of Health and Care Excellence
NIHSS: National Institute of Health Stroke Scale
PITeS-TIiSS: Telemedicine and eHealth Innovation Platform: Information Communications Technology for Research and Information Challenges in Health Services
SNOMED CT: Systematized Nomenclature of Medicine Clinical Terms
VRUH: Virgen del Rocío University Hospital
WHO: World Health Organization
