# Correction: A Personalized Ontology-Based Decision Support System for Complex Chronic Patients: Retrospective Observational Study

**DOI:** 10.2196/46102

**Published:** 2023-02-28

**Authors:** Esther Román-Villarán, Celia Alvarez-Romero, Alicia Martínez-García, German Antonio Escobar-Rodríguez, María José García-Lozano, Bosco Barón-Franco, Lourdes Moreno-Gaviño, Jesús Moreno-Conde, José Antonio Rivas-González, Carlos Luis Parra-Calderón

**Affiliations:** 1 Computational Health Informatics Group, Institute of Biomedicine of Seville Virgen del Rocío University Hospital, Consejo Superior de Investigaciones Científicas University of Seville Seville Spain; 2 Primary Care Camas Clinical Management Unit Seville Spain; 3 Internal Medicine Department Virgen del Rocío University Hospital Seville Spain

In “A Personalized Ontology-Based Decision Support System for Complex Chronic Patients: Retrospective Observational Study” (JMIR Form Res 2022;6(8):e27990) the authors made three changes.

Affiliation 1 originally appeared as:

Group of Research and Innovation in Biomedical Informatics, Biomedical Engineering and Health Economy, Institute of Biomedicine of Seville (IBiS), Virgen del Rocío University Hospital, University of Seville, Seville, Spain

But has been changed to:

Computational Health Informatics Group, Institute of Biomedicine of Seville, Virgen del Rocío University Hospital, Consejo Superior de Investigaciones Científicas, University of Seville, Seville, Spain

Additionally, the text under “Acknowledgments” has been changed from:

This research was partly funded by the Innovation in Medical Technologies and Health (ITEMAS; platform for the dynamization and innovation of the industrial capacities of the National Health System) platform (code #PT13/0006/0036 and #PT17/0005/0036) and by the PITeS-TIiSS project (code #PI15/01213) from the Carlos III National Health Institute, both cofunded by the European Regional Development Fund (ERDF). We would like to thank Dr Rosario Villarán Martínez for the help during the validation phase of the PITeS-TIiSS tool.

To read as follows:

This research was partly funded by the Innovation in Medical Technologies and Health (ITEMAS; platform for the dynamization and innovation of the industrial capacities of the National Health System) platform (code #PT13/0006/0036 and #PT17/0005/0036) and by the PITeS-TIiSS project (code #PI15/01213) and Smart-PITeS project (code #PI18/00700) from the Carlos III National Health Institute, both cofunded by the European Regional Development Fund (ERDF). We would like to thank Dr Rosario Villarán Martínez for the help during the validation phase of the PITeS-TIiSS tool.

The correction will appear in the online version of the paper on the JMIR Publications website on February 28, 2023, together with the publication of this correction notice. Because this was made after submission to PubMed, PubMed Central, and other full-text repositories, the corrected article has also been resubmitted to those repositories.

